# Variants of Novel Immunomodulatory Fc Receptor Like 5 Gene Are Associated With Multiple Sclerosis Susceptibility in the Polish Population

**DOI:** 10.3389/fneur.2021.631134

**Published:** 2021-04-06

**Authors:** Monika Chorazy, Natalia Wawrusiewicz-Kurylonek, Edyta Adamska-Patruno, Agata Czarnowska, Olga Zajkowska, Katarzyna Kapica-Topczewska, Renata Posmyk, Adam Jacek Kretowski, Jan Kochanowicz, Alina Kułakowska

**Affiliations:** ^1^Department of Neurology, Medical University of Bialystok, Bialystok, Poland; ^2^Department of Endocrinology, Diabetology and Internal Medicine, Medical University of Bialystok, Bialystok, Poland; ^3^Department of Clinical Genetics, Medical University of Bialystok, Bialystok, Poland; ^4^Clinical Research Centre, Medical University of Bialystok, Bialystok, Poland; ^5^Faculty of Economic Sciences, University of Warsaw, Warsaw, Poland

**Keywords:** multiple sclerosis, FCRL5 receptors, genetic variants, rs2012199, rs6679793

## Abstract

Fc receptors have been shown to play a role in several autoimmune diseases. We aimed to test, for the first time, whether some of the single nucleotide variants in the *FCRL5* gene were associated with multiple sclerosis (MS) susceptibility and clinical manifestations in the Polish population. The case-control study included 94 individuals with MS and 160 healthy subjects. We genotyped two single nucleotide variants of the *FCRL5* gene: rs2012199 and rs6679793. The age of onset, disease duration, and clinical condition of the MS subjects were analyzed. For statistical analysis, we used the chi-squared test confirmed with Fisher's exact test. We observed the significant differences in the distribution of investigated *FCRL5* genotypes between MS subjects and healthy controls. The CC and CT genotypes, as well as the C allele of rs2012199, were significantly more common in the MS subjects, as were genotypes AA and AG, and allele A of rs6679793. We noted that decreased MS susceptibility was associated with the T allele rs2012199 (OR = 0.37, *p* = 0.0002) and G allele rs6679793 (OR = 0.6, *p* = 0.02). Our results support the role of the *FCRL5* locus in MS predisposition and extend the evidence of its influence on autoimmunity.

## Introduction

Autoimmune diseases (AIDs) result from an impaired immune response, and their development includes a strong heritable component (1); multiple sclerosis (MS) involves autoimmune responses to myelin antigens. The occurrence of MS may be promoted by certain genetically predetermined mechanisms combined with other genetic and/or environmental factors associated with specific diseases (2); however, little is known about the role of peripheral blood cells in this neurodegenerative component. Many studies point toward the role of T-cells in mediating inflammatory damage within the central nervous system, and MS has already been regarded as a T-cell-mediated disorder (3). In addition, genetic loci that have been identified close to immunologically relevant genes are significantly overrepresented among MS subjects, thus implicating the T-cell differentiation in the pathogenesis of MS (4). However, raising the inflammatory process to the level of systemic and clinical manifestations involves not only the T-cells but also the whole amplifying and disease-perpetuating complex of cellular-network factors, triggering the production of cytokines and other paracrine mediators. These factors include B-cells (5).

Despite the established importance of B-cells in the pathogenesis of autoimmunity, the immune mechanisms that underlie their role have not been completely defined so far (6). It is known that the B-cell pathway plays many important roles other than as precursors of terminally differentiated, antibody-secreting plasma cells, and their additive role in the pathogenesis of MS is being investigated (7, 8). Evidence supports important B-cell roles in MS development; disease suppression, reduction in neurodegeneration, and changes in disability progression have been observed (9) under B-cell depleting therapies, such as treatment with ocrelizumab, that have received the first FDA indication for primary progressive MS treatment (10).

The activation of B-cells and the mediation of the specific recognition of antigens by leukocytes are promoted by a large family of Fc receptors (11). Human FCRL5 (Fc receptor-like protein 5) is a novel IgG receptor of the immunoglobulin superfamily (IgSF), and its expression is mainly restricted to B-cells (12). Genome-wide screening has revealed that the entire 1q21-q23 region—which includes the *FCRL5* gene—has been implicated in several AIDs; in subjects with high neurodegeneration, some changes in the peripheral blood mononuclear cells (PBMCs), including lower *FCRL5* expression and higher peripheral blood B-cells activation status—characterized by a down-regulation of B-cell-specific genes—have been noted (13). Therefore, we aimed to investigate whether two common SNVs of the *FCRL5* gene may contribute to the development of MS and its clinical manifestation in the Polish population.

## Methods

### Ethics

The study was approved by the Bioethical Committee of Medical University of Bialystok, Poland (R-I-002/334/2018) and was conducted in accordance with the Helsinki Declaration. All participants signed informed consent forms before enrollment in the study.

### Participants

We genotyped 254 subjects: 94 with MS (47 males and 47 females, mean age 41.15 ± 9.74 years, Table 1) and 160 unrelated healthy adults (85 males, 75 females; mean age 37.6 ± 2.16 years) for two SNVs of the *FCRL5* gene (rs2012199, rs6679793). Participants from both groups were recruited from the same ethnic group and geographical area, which has been previously described in detail (14–17). In the control group, we enrolled individuals without any family history of autoimmune diseases. The clinical characteristics of the MS study population are presented in Table 1.

**Table 1:** The clinical characteristic of the MS subjects' group.

**Characteristic**	**MS subjects,** ***n* = 94**	**Women,** ***n* = 47**	**Men,** ***n* = 47**
Age at onset (years)	41.15 ± 0.79	42.78 ± 0.98	37.14 ± 1.11
Disease duration (years)	8.12 ± 0.42	8.18 ± 0.51	7.96 ± 0.75
EDSS	1.91 ± 0.10 1.5 (IQR 1-3)^*^	1.99 ± 0.19 2 (IQR 1-3)^*^	1.83 ± 0.12 1 (IQR 1-3)^*^

*Data are presented as mean ± SD*.

* *For EDSS, median values and IQRs are presented. MS, multiple sclerosis; EDSS, Expanded Disability Status Scale; IQR, interquartile range*.

### Clinical Disease Manifestation

The ages of onset and disease duration were analyzed. The clinical condition of MS subjects was evaluated—at the time of diagnosis, before providing any treatment—using the Kurtzke Expanded Disability Status Scale (EDSS).

### Genetic Analysis

Genomic DNA was isolated using the Qiagen column separation method (QIAamp DNA Blood Mini Kit, Qiagen, Germany), according to the manufacturer's instructions. The purity and concentration of the obtained preparations were evaluated by spectrophotometry using the Nano Drop 2000 device (ThermoFisher Scientific, USA). All DNA samples were normalized to 50 ng/μl. The SNVs were tested in duplicates using the QuantStudio 12K Flex platform on OpenArray plates (ThermoFisher Scientific, USA), with TaqMan molecular probes applied to the plates, and the TaqMan Genotyping Master Mix Protocol (ThermoFisher Scientific, USA). We analyzed two SNVs in the *FCRL5* gene, rs2012199, and rs6679793. We used a sample without a template as a negative control to measure any false positive signal caused by molecular contamination. The initial analysis of real-time PCR data was performed using the TaqMan Genotyper genotyping data analysis software (Thermo Fisher Scientific).

### Statistical Analysis

All calculations were performed using R version 4.0.2(18). Because of the relatively small sample size, Fisher's exact test was used to compare genotypic frequencies of rs2012199 SNVs between the two groups, and the odds ratios (ORs) were calculated with a Haldane-Anscombe correction. Otherwise, a chi-square test was used for all 2 × 2 and 2 × 3 contingency tables. We reported the *p*-values and OR (or corrected OR), together with their 95% confidence intervals (CIs). The linkage disequilibrium (LD) was checked using the chi-square test. The statistical significance level for all two-sided tests was set at *p* < 0.05.

## Results

The frequencies of the investigated genotypes and alleles in the studied groups are presented in Table 2.

**Table 2:** The genotypes and allele frequencies of the investigated SNVs of *FCRL5* gene, in the studied groups.

**SNV**	**Genotypes/Allele**	**MS (*****n*** **=** **94)**		**Control (*****n*** **=** **160)**		***P***	**OR (95% CI)**
		***n***	**Frequency**	***n***	**Frequency**		
rs2012199	CC	11	(11.70%)	0	(0.00%)		
	CT	19	(20.21%)	30	(18.75%)	0.0002^*^	0.03 (0.00–0.28)^**^
	TT	64	(68.09%)	130	(81.25%)	≤0.001^*^	0.02 (0.00–0.19)^**^
	C	41	(0.22)	30	(0.09)		
	T	147	(0.78)	290	(0.91)	0.0002	0.37 (0.22–0.64)
rs6679793	AA	11	(11.70%)	5	(3.12%)		
	AG	32	(34.04%)	52	(32.50%)	0.0292	0.28 (0.07–0.98)
	GG	51	(54.26%)	103	(64.38%)	0.0115	0.23 (0.06–0.76)
	A	54	(0.29)	62	(0.19)		
	G	134	(0.71)	258	(0.81)	0.0207	0.6 (0.38–0.93)

* *For the rs2012199 genotypes we reported the p-values of the Fisher's exact test; otherwise, chi-squared tests were used*.

** *Haldane–Anscombe corrected ORs. SNV, single nucleotide variant; MS, multiple sclerosis; OR, odds ratio*.

LD analysis of rs2012199 and rs6679793 showed that investigated SNPs were not in linkage disequilibrium (D' = 0.315, *r*^2^ = 0.061, χ^2^ = 0.15, df = 1, *p*-value 0.698). The allele frequencies of the homozygous major, minor, and heterozygous SNVs agreed with the Hardy–Weinberg equilibrium.

Fisher's exact test was used to determine whether there was any evidence of heterogeneity between the study groups in the three rs2012199 genotypes. The proportion of MS cases was not equally distributed across genotypes, with *p* < 0.0001. The ORs for MS in CT vs. CC as well as TT vs. CC genotypes were further studied using the Haldane–Anscombe correction. The CC genotype of rs2012199 has been identified only in individuals with MS. The corrected OR of MS in subjects with the CT genotype was equal to 0.03 (95% CI 0.00–0.28, *p* = 0.0002) and 0.02 (95% CI 0.00–0.19, *p* < 0.0001) in subjects with the TT genotype. We also observed similar associations for the allele distribution; allele C was significantly more common in MS subjects (0.22 vs. 0.09). The OR of MS was significantly lower in the T allele group (OR = 0.37, 95% CI of OR 0.22–0.64, chi-square test for association *p* = 0.0002).

Similar results were obtained for the genotypes and alleles of rs6679793. The heterogeneity of the study groups was compared in three rs6679793 genotypes using the chi-square test, and there was evidence that the proportion of MS cases was not equally distributed across genotypes with *p* = 0.0187. The OR of MS in subjects with the AG genotype was equal to 0.28 (95% CI 0.07–0.98, *p* = 0.0292) and 0.23 (95% CI 0.06–0.76, *p* = 0.0115) in subjects with the GG genotype. Allele A was significantly more common in MS subjects (0.29 vs. 0.19). The OR of MS was significantly lower in the G allele group (OR=0.6, 95% CI of OR 0.38–0.93, chi-square test for association *p* = 0.0207).

We did not notice any differences in the age of onset, duration of disease, or EDSS between the studied genotypes (data not shown).

## Discussion

We investigated two exonic non-synonymous SNVs in the coding region of the *FCRL5* gene (located at chromosome 1). The first was rs6679793, situated at bp 157544307 A>G (GRCh38.p12), that codes for an amino acid change of tyrosine to histidine at position 267 (p.Tyr267His). The second was rs2012199, at bp 157539235 C>T (GRCh38.p12), that codes for an amino acid change from glycine to aspartate at position 418 (p.Gly418Asp). To the best of our knowledge, the functional effects of these amino acid-substituting variations on protein structure and function have not been investigated so far. Nevertheless, considering the functional role of FCRL5 protein (19), the genetic variations in *FCRL5* gene may affect the proliferation and switched isotype expression of B cells and may play a role in the expansion and development of antigen-primed cells. Our study showed that genotypes CC, CT, and allele C of rs2012199—as well as genotypes AA, AG, and allele A of rs6679793 of the *FCRL5* gene—are more common in the subjects with MS, whereas allele T of rs2012199 and allele G rs6679793 seemed to be protective genetic variants against the MS risk. We did not observe any associations between the SNVs studied and MS clinical manifestations investigated.

*FCRL* genes have been recently reported to play a significant role in AIDs; they are involved in the development of rheumatoid arthritis (RA), autoimmune thyroid disease (AITD), Graves' disease, and Behçet's disease (20–22). Studies indicating the role of FCRL receptors and their genes in the AIDs mostly focus on the FCRL3 family, which modulates the regulatory T-cell activity (23) and plays an important role in MS pathogenesis (24). It has been noted that the *FCRL3* gene confers a risk of allergic rhinitis (an autoimmune condition) in the Chinese population (25). *FCRL3* is also involved in protection against MS (26). High *FCRL3* expression has been found in B-lymphocytes and augmented autoantibody production in individuals with the rheumatoid arthritis-susceptible genotype (27). We know less about the genetic variants of the other subtypes of FCRL receptors, such as FCRL5 and their associations with AIDs. It has been noted that FCRL5 is differentially bound to all IgG isotypes (28) and is preferentially expressed widely among naive plasma B-cells, memory B-cells, and plasma cells but also at low levels in pre-B cells. FCRL5 may be involved in B-cell development and differentiation (19), and due to inhibition of a BCR-mediated response, it may play an important immunomodulatory role; it may also be a useful marker of B-cell stages (20, 29, 30). As concluded by Rostamzadeh et al. (20), the FCRLs appear to be logical candidates for the development of antibody- and cell-based immunotherapy approaches for B-cell malignancies and immune-mediated diseases, but also attractive targets for immunodiagnostic approaches, disease monitoring, and clinical prognosis. This has been strongly supported by a recently published study by Owczarczyk et al. (31). However, the rs2012199 and rs6679793 SNVs of the *FCRL5* gene investigated in our study have been associated only with an autoimmune thyroid disease so far (21). This is despite the fact that they represent a potential biomarker of clinical response for rituximab in rheumatoid arthritis (31) and the observation that *FCRL5* gene expression is strongly upregulated on the bone marrow plasma cells of multiple myeloma and Burkitt Lymphoma (20).

To the best of our knowledge, we report for the first time that rs2012199 and rs6679793 SNVs of the *FCRL5* gene may be associated with MS susceptibility. We observed a significantly increased prevalence of the investigated SNVs and alleles of the *FCRL5* gene in the MS subjects. Nevertheless, our observations must be confirmed in the larger populations and different ethnicities, since our study was conducted in a relatively small study group; this is the major limitation of our study. Another limitation is the fact that we have investigated only two SNVs of the *FCRL5* gene, and the impact of variations in other closely located genes cannot be excluded. A strong linkage disequilibrium (LD) across the neighboring genes has been previously observed (32). Moreover, Simmonds et al. (33) concluded that in the UK population, an association of other autoimmune disease—such as Graves' disease—with the *FCRL5* SNPs was secondary to a linkage disequilibrium with the *FCRL3* SNPs. Therefore, a refined association study in the entire region is required to determine the associations between genetic variants and haplotypes in the context of an MS risk. Nevertheless, our results suggest that polymorphisms in the *FCRL5* gene may be a risk factor for autoimmunity, and that FCRL5-dependent mechanisms may be involved in MS development. Further functional studies are needed to establish the functional nature of the investigated genetic variants, which may predispose to MS development.

## Data Availability Statement

The datasets presented in this article are not readily available in order to protect the patients' privacy as written informed consent for the publication of this data was not obtained. Requests to access the datasets should be directed to Dr. Monika Chorazy (chorazym@op.pl).

## Ethics Statement

The studies involving human participants were reviewed and approved by Bioethical Committee of Medical University of Bialystok, Poland. The patients/participants provided their written informed consent to participate in this study.

## Author Contributions

MC and NW-K: conceptualization and methodology. OZ: formal analysis. MC, NW-K, AC, KK-T, and RP: investigation. MC and EA-P: writing—original draft preparation, writing—review, and editing. AJK, JK, and AK: supervision. MC: project administration and funding acquisition. All authors have read and agreed to the published version of the manuscript.

## Conflict of Interest

The authors declare that the research was conducted in the absence of any commercial or financial relationships that could be construed as a potential conflict of interest.
